# A Novel Framework for Whole-Slide Pathological Image Classification Based on the Cascaded Attention Mechanism

**DOI:** 10.3390/s25030726

**Published:** 2025-01-25

**Authors:** Dehua Liu, Bin Hu

**Affiliations:** 1 Gansu Provincial Key Laboratory of Wearable Computing, School of Information Science and Engineering, Lanzhou University, Lanzhou 730000, China; liudh21@lzu.edu.cn; 2 School of Medical Technology, Beijing Institute of Technology, Beijing 100081, China

**Keywords:** tumor diagnosis, attention mechanism, whole-slide images, computer-aided diagnosis

## Abstract

This study introduces an innovative deep learning framework to address the limitations of traditional pathological image analysis and the pressing demand for medical resources in tumor diagnosis. With the global rise in cancer cases, manual examination by pathologists is increasingly inadequate, being both time-consuming and subject to the scarcity of professionals and individual subjectivity, thus impacting diagnostic accuracy and efficiency. Deep learning, particularly in computer vision, offers significant potential to mitigate these challenges. Automated models can rapidly and accurately process large datasets, revolutionizing tumor detection and classification. However, existing methods often rely on single attention mechanisms, failing to fully exploit the complexity of pathological images, especially in extracting critical features from whole-slide images. We developed a framework incorporating a cascaded attention mechanism, enhancing meaningful pattern recognition while suppressing irrelevant background information. Experiments on the Camelyon16 dataset demonstrate superior classification accuracy, model generalization, and result interpretability compared to state-of-the-art techniques. This advancement promises to enhance diagnostic efficiency, reduce healthcare costs, and improve patient outcomes.

## 1. Introduction

With the global population aging and lifestyle shifts, cancer has become a significant health concern, its incidence rising steadily worldwide. Pathological diagnosis, deemed the “gold standard” for tumor confirmation [1], necessitates the meticulous examination of stained tissue section images to assess cancer cell presence and activity. However, this manual process, heavily reliant on pathologist expertise, is time-consuming and labor-intensive, struggling to cope with the surging volume of pathological slides. Moreover, the inherent complexity of whole-slide images presents substantial diagnostic challenges [2,3]. Consequently, enhancing the automation and accuracy of pathological diagnosis has become a pivotal research focus.

Despite deep learning’s substantial advancements in image recognition and classification, its application in pathological image analysis encounters several hurdles. Firstly, whole-slide images (WSIs) exhibit large data volumes and high resolution, imposing significant demands on image processing. Secondly, the heterogeneity and complexity of these images require models to adeptly capture subtle variations. Lastly, the high annotation costs limit the availability of training data for deep learning models. Thus, designing efficient models capable of processing large data volumes while capturing fine details is imperative. Recently, multiple instance learning (MIL) [4] has emerged as a dominant paradigm to tackle these challenges [5,6,7,8,9,10,11,12,13,14,15,16,17]. MIL application in WSI analysis involves three key steps: (1) segmenting WSIs into manageable patches (e.g., 256 × 256 pixels); (2) extracting features from these patches; and (3) aggregating features to form a slide-level representation, facilitating classification. Attention-based MIL frameworks have recently gained widespread adoption for WSI classification [18,19,20,21,22,23].

**Figure 1:**
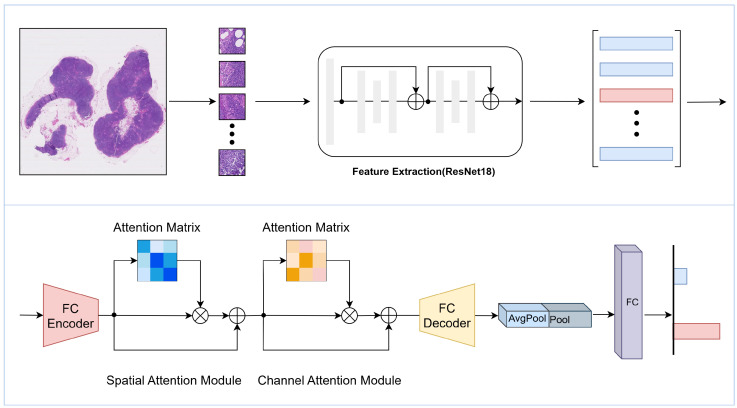
Overview of the proposed CAMIL framework. The framework involves segmenting whole-slide images into patches, extracting features using a pre-trained ResNet18 model, and processing the feature vector matrix through an encoder, attention modules, decoder, pooling layer, and fully connected layer to generate the final prediction.

The primary aim of this research is to elevate the precision and efficiency of pathological image analysis through the creation of a groundbreaking deep learning framework. As illustrated in Figure 1, our framework is equipped with a sophisticated cascaded attention mechanism, designed to adeptly extract pivotal features from both the spatial and channel dimensions of pathological images. This innovative approach not only refines the detection of cancer cells but also enriches diagnostic insights, thereby enhancing the overall interpretability of the analysis.

Our primary contributions are as follows:

1. The introduction of a novel cascaded attention mechanism that synergistically merges spatial and channel attention to refine feature recognition in pathological images.
2. The design of an efficient deep learning model optimized for handling extensive WSI data while preserving high accuracy and minimizing computational overhead.
3. Extensive experimental validation on the CM16 benchmark dataset, showcasing the superior performance of our model compared to existing top models.
4. A thorough analysis of model interpretability using Grad-CAM [24], fostering confidence and understanding among clinicians for automated pathological diagnosis.

The remainder of this paper is organized as follows: Section 2 offers an overview of the current state of research in the application of multiple instance learning (MIL) to the classification of whole-slide images (WSIs), along with the utilization of attention mechanisms within MIL. Section 3 provides a detailed exposition of the MIL framework and outlines the constituent elements of the proposed cascaded attention-based multiple instance learning (CAMIL) model. Section 4 presents the experimental results and engages in an in-depth analysis of the obtained findings. This paper culminates in a summary in Section 5.

## 2. Related Work

### 2.1. Advanced Techniques in Medical Image Classification

Recent advancements in deep learning have significantly improved the capabilities of image classification, particularly in the domain of medical imaging. Key innovations such as adversarial training, transfer learning, and graph convolutional aggregation have been developed to address critical challenges, including class imbalance, low inter-class variation, and complex data relationships.

Adversarial Training and Transfer Learning: These techniques have proven effective in tackling issues like class imbalance and low inter-class variation, which are common in datasets such as those for skin lesion analysis [25]. A proposed two-stage framework synthesizes under-represented class samples using unpaired image-to-image translation, followed by training a deep convolutional neural network classifier on the augmented dataset. This approach not only balances the dataset but also enhances model performance by minimizing focal loss, leading to significant improvements in sensitivity and the area under the curve (AUC) compared to baseline methods. These results highlight the potential of integrating adversarial training and transfer learning into medical image classification tasks.

Graph Convolutional Aggregation: Another notable advancement is the application of graph convolutional networks (GCNs) to capture intricate relationships between data points [26]. By incorporating skip connections and identity mapping, GCNs effectively mitigate the oversmoothing problem that often arises in such models. This enhancement leads to improved classification accuracy and robustness, demonstrating the utility of graph-based approaches in handling complex medical data.

While these advanced techniques have substantially enhanced medical image classification, certain challenges remain unresolved—particularly the issue of weakly labeled data in pathological images. To address this limitation, multiple instance learning (MIL) has emerged as a promising solution.

### 2.2. Multiple Instance Learning: A Targeted Approach for Pathological Image Classification

Multiple instance learning (MIL) is a specialized machine learning framework designed to handle scenarios where only coarse or partial labeling information is available. It organizes data into bags of instances and performs learning at the bag level, enabling models to manage uncertain or incompletely annotated data—a crucial requirement in medical image analysis. In the context of pathological image analysis, where detailed annotations are typically time-consuming and costly, MIL offers a practical and efficient solution.

MIL models can be broadly categorized into two types: instance-level methods [27,28,29,30,31] and embedding-level methods [18,19,20,32,33]. Instance-level methods aim to classify individual instances within a bag and aggregate their predictions to make bag-level decisions. In contrast, embedding-level methods focus on aggregating instance representations to form a unified bag representation, which is then used for classification. Although instance-level methods are conceptually straightforward, embedding-level methods generally deliver more stable and accurate performance [33,34]. Consequently, this study adopts an embedding-level approach to better address the inherent complexity and variability of pathological images.

Despite its advantages, traditional MIL methods often fail to account for the varying importance of instances within a bag. This limitation motivates the adoption of attention mechanisms, which are discussed in the following section.

### 2.3. Enhancing Multiple Instance Learning with Attention Mechanisms

Traditional multiple instance learning (MIL) methods typically overlook the varying significance of instances within a bag, thereby failing to fully exploit critical information. To overcome this limitation, attention-based MIL (AB-MIL) [18] was introduced, assigning attention scores to instances to emphasize those most relevant for classification. By leveraging weighted averaging based on attention scores, AB-MIL generates more informative bag representations, resulting in greater flexibility and improved accuracy in pathological image classification.

Recent developments in attention-based MIL methods have further boosted performance in pathological image classification [19,20,33,35]. Building upon these advancements, our research introduces a novel cascaded attention mechanism. This strategy refines attention allocation at the instance level while introducing a global attention score at the bag level, facilitating a deeper understanding of complex inter-instance relationships. The proposed cascaded attention mechanism significantly improves pathological image classification by precisely identifying and weighting key features, addressing critical challenges in medical image analysis.

In summary, the integration of advanced deep learning techniques, tailored frameworks such as MIL, and innovative attention mechanisms underscores the potential to tackle persistent challenges in medical image analysis. These methodologies collectively provide a robust foundation for advancing the field of pathological image classification.

## 3. Method

In the present study, we outline our method for the weakly supervised classification of whole-slide images (WSIs). This section aims to explore the conceptual framework of multiple instance learning (MIL) and elaborate on our innovative model, termed CAMIL.

### 3.1. Background: MIL Formulation

Multiple instance learning (MIL) represents a supervised learning technique particularly suited for situations where the labeling is conducted at the bag level, rather than targeting individual instances. In contrast to the conventional supervised learning approach, where each sample is directly linked to a single label, MIL involves bags that encompass numerous instances, with the label assigned to the bag as an entity. In the MIL paradigm, we postulate a dataset $D=\{\left(X_{1},Y_{1}\right),\left(X_{2},Y_{2}\right),\ldots ,\left(X_{N},Y_{N}\right)\}$, comprising bag–label pairs $\left(X_{i},Y_{i}\right)$. Each bag $X_{i}$ is a compilation of instances, represented by $X_{i}=\left\{x_{i1},x_{i2},\ldots ,x_{in}\right\}$. In this context, $x_{ij}$ indicates the *j*-th instance within the bag $X_{i}$, and *n* signifies the total number of instances contained within $X_{i}$. Each bag $X_{i}$ is paired with a label $Y_{i}$, which, in the context of binary classification, is usually a member of the set {0,1}.

The principle of multiple instances posits that in a bag that is positively labeled $Y_{i}=1$, there is at least one positive instance present. On the other hand, if a bag is labeled negatively $Y_{i}=0$, it is assumed that all instances within that bag are negative. This principle can be articulated in a formal manner as follows:

(1) $$Y_{i}=\left\{\begin{array}{cc} 1, & \;\mathrm{if}\;\exists x_{ij}\in X_{i}\;\mathrm{such}\;\mathrm{that}\;x_{ij}\;\mathrm{is}\;\mathrm{positive}\;\\ 0, & \;\mathrm{if}\;\forall x_{ij}\in X_{i},x_{ij}\;\mathrm{is}\;\mathrm{negative}\; \end{array}\right.$$

The central difficulty inherent in this approach stems from the opacity of individual instance labels. As such, it is imperative to identify those instances that most profoundly influence the bag’s label. To tackle this issue, multiple instance learning (MIL) algorithms are compelled to develop an aggregation strategy that consolidates instance-level information to yield a prediction at the bag level. Typically, this is achieved by employing an instance-level classifier *f*, followed by an aggregation function *g* that merges the classifier’s scores from the instances to determine the bag’s label prediction. For instance, a straightforward aggregation technique could be to select the highest instance score as the predictive score for the bag:

(2) $${\hat{Y}}_{i}=g\left(\left\{f\left(x_{ij}\right)|x_{ij}\in X_{i}\right\}\right)=maxf\left(x_{ij}\right)$$

In the paradigm of attention-based multiple instance learning (AB-MIL), the aggregation function *g* takes the form of an attention mechanism, which allocates a weight $w_{ij}$ to each instance $x_{ij}$. These weights encapsulate the relative significance of each instance in contributing to the prediction of the bag’s label. Subsequently, a weighted average is calculated, leveraging these weights, to generate the predictive score for the bag:

(3) $${\hat{Y}}_{i}=\sum_{j}w_{ij}\cdot f\left(x_{ij}\right)$$

Each weight $w_{ij}$ is generally determined via a trainable attention function, and the weights corresponding to each bag are commonly normalized to ensure that the sum across all instances within the bag equals unity, i.e., $\sum_{j}w_{ij}=1$.

This methodology enables the MIL framework to infer the significance of instances indirectly through the bag label, obviating the need for direct observation of the labels of the individual instances. It has demonstrated remarkable efficacy across diverse domains such as medical imaging, text categorization, and audio signal processing, especially in contexts where annotation is either expensive or challenging to secure.

### 3.2. Cascaded Attention Mechanism

In light of the numerous challenges faced in the domain of multi-instance classification, this study, drawing inspiration from the insights presented in [36], introduces an innovative framework. At its core is the incorporation of a cascaded attention mechanism module, which has been meticulously crafted to capture contextual information across both spatial and channel dimensions. Considering the inherent heterogeneity of tumors and the inevitable errors inherent in the production of whole-slide pathology images, even images originating from the same tumor type can exhibit considerable diversity in terms of color and micro-features. It is this diversity that subtly introduces intra-class variability, which in turn adversely affects classification accuracy. To surmount this challenge, our framework integrates attention mechanisms at both the spatial and channel levels to glean global contextual information, thereby significantly enhancing the recognition accuracy of pathology images.

**Figure 2:**
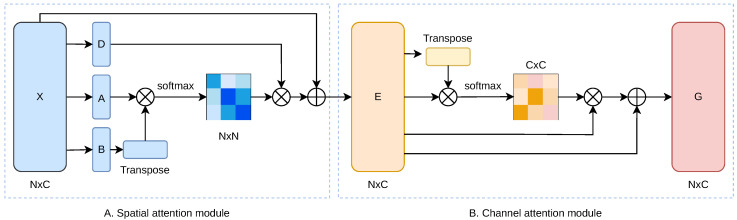
An overview of the cascaded attention mechanism is provided. As depicted in the figure, the details of the two sub-modules within the cascaded attention mechanism, the position attention module and the channel attention module, are illustrated in (**A**) and (**B**), respectively.

As depicted in Figure 2, the framework employs a dual-module cascaded approach, wherein spatial and channel attention modules collaborate to refine the quality of feature representation for pixel-level predictions. The preprocessed pathology image feature matrix is fed into these two cascaded attention modules. This procedure yields a novel feature matrix, enriched with contextual information gleaned from the pathology images, through a series of three deliberate steps.

The first step involves constructing a spatial attention matrix that encapsulates the spatial interdependencies among patches within the feature matrix. This attention matrix is then merged with the original feature matrix through matrix multiplication. In the subsequent step, the resultant feature matrix is fused with the initial feature matrix using an element-wise addition operation to create the ultimate feature representation.

The channel attention module functions analogously to the spatial module, but with a focus on the channel dimension, thereby generating a corresponding channel attention matrix. The refined processing afforded by these two cascaded attention modules culminates in a final feature representation that serves as a more resilient basis for the precise prediction of pathology images.

### 3.3. Spatial Attention Module

Accurate and distinctive feature representation is pivotal to the efficacy of pathological image classification. This representation is typically derived from the adept capture of the wealth of contextual information embedded within whole-slide images. In pursuit of a comprehensive exploration and modeling of the complex contextual relationships enveloping local features, we have developed and incorporated a spatial attention module. The objective of this module is to imbue local features with a rich tapestry of contextual information, thereby markedly augmenting their representational power. The subsequent sections will delineate the method by which the spatial attention module assiduously compiles spatial contextual information and the manner in which this contributes to the optimization of our model’s performance.

As represented in Figure 2, we commence by feeding a feature vector matrix $X\in R^{N\times C}$ into a linear layer. Specifically, this process involves projecting the input matrix *X* into two distinct feature spaces using two separate sets of learnable parameters. Mathematically, the generation of the two novel feature matrices *A* and *B*, with $\left\{A,B\right\}\in R^{N\times C}$, can be expressed as:

(4) $$A=XW_{A}+b_{A},\;B=XW_{B}+b_{B}$$

where $W_{A},W_{B}\in R^{C\times C}$ are the weight matrices, and $b_{A},b_{B}\in R^{C}$ are the corresponding bias vectors. Here, *N* represents the number of samples (rows), while *C* denotes the channel size of each feature vector (columns). Subsequently, we perform matrix multiplication between the *A* and $B^{T}$, followed by the application of a softmax layer to transform the resultant matrix into a spatial attention matrix $S\in R^{N\times N}$:

(5) $$s_{ji}=\frac{\exp\left(A_{i}\cdot B_{j}\right)}{\sum_{i=1}^{N}\exp\left(A_{i}\cdot B_{j}\right)}$$

In this context, $s_{ji}$ signifies the degree of influence that the patch located at position *i* exerts on the patch at position *j*. Patches that exhibit a higher degree of similarity are deemed more interconnected. In parallel, the feature vector matrix *X* is processed through an additional linear layer to yield a novel feature vector *D*. Following this, matrix multiplication is conducted between the transpose of the spatial attention matrix *S* obtained earlier and *D*. The product is then scaled by a trainable scalar α and undergoes an element-wise addition with *X*, culminating in the production of the output matrix $E\in R^{N\times C}$:

(6) $$E_{j}=\alpha \sum_{i=1}^{N}\left(s_{ji}D_{i}\right)+X_{j}$$

The variable α denotes an adaptable weight parameter, initially set to zero. Analyzing the presented formulation, it becomes evident that the features for each patch are computed via a weighted amalgamation of the features from all other patches. This process of weighted integration guarantees that the resultant output conveys both the nuanced details of individual local regions and the overarching contextual information of the pathological image. By adopting this strategy, the model is equipped to grasp the image’s global structure, thereby refining its proficiency in the identification and interpretation of pathological areas.

### 3.4. Channel Attention Module

When constructing feature vectors, each channel encapsulates a distinct representation of a patch with respect to particular characteristics. These channels are not autonomous entities; rather, they are interconnected through a network of complex relationships. Acknowledging this interconnectedness, we introduce a method that markedly augments the representation of patch features by emphasizing the most informative aspects of channel features. To accomplish this objective, we have meticulously crafted a channel attention mechanism capable of precisely identifying and highlighting these interdependencies. This mechanism refines the feature extraction process, directing the model’s focus toward the most significant channels, which in turn boosts the overall efficacy of feature representation.

As depicted in Figure 2, the output $E\in R^{N\times C}$ from the spatial attention module serves as the input for this subsequent phase. We proceed by performing matrix multiplication between the $E^{T}$ and *E*, and then apply a softmax layer to derive the channel attention matrix $C\in R^{C\times C}$:

(7) $$c_{ji}=\frac{\exp\left(E_{i}\cdot E_{j}\right)}{\sum_{i=1}^{N}\exp\left(E_{i}\cdot E_{j}\right)}$$

Within this framework, $c_{ji}$ quantifies the degree to which channel *i* affects channel *j*. Moreover, we multiply the matrix $C^{T}$ with matrix *E*, followed by scaling the resultant matrix by a scalar β. An element-wise addition is then performed between this scaled matrix and *E*, culminating in the derivation of the final output matrix *G*, which retains the same dimensionality of $R^{N\times C}$.

(8) $$G_{j}=\beta \sum_{i=1}^{N}\left(c_{ji}E_{i}\right)+E_{j}$$

The parameter β here denotes an initializable learnable weight, set to zero at the outset. The preceding formulation reveals that the ultimate feature for each channel is computed as a result of a weighted integration with the features of all other channels. This method of weighted integration markedly elevates the discriminative capacity of pathological image features, enabling more precise characterization in the examination of intricate tissue architectures.

In order to optimize the harnessing of contextual information, we have embraced a tactic that amalgamates features derived from two distinct attention modules. This tactic specifically entails concatenating the outputs of these two modules to realize the final feature amalgamation.

## 4. Experiments and Results

In this segment, we undertake an exhaustive comparative analysis of our proposed methodology against other leading cutting-edge techniques in the field. Additionally, we carry out a series of rigorous ablation studies to elucidate the substantial impact that each constituent of our framework has on the aggregate predictive efficacy.

### 4.1. Dataset

Our experimental findings are documented utilizing the extensively benchmarked Camelyon16 and TCGA Lung Cancer public dataset. As a premier resource for whole-slide image (WSI) classification research, Camelyon16 comprises 399 WSIs intended for breast cancer screening, each annotated as either normal or tumor. The dataset’s meticulous pixel-level annotations of positive WSIs render it apt for both classification and region of interest (ROI) localization tasks [35]. The TCGA Lung Cancer dataset consists of two subtypes: lung adenocarcinoma and lung squamous cell carcinoma, with a total of 1054 diagnostic digital slides available for download from the National Cancer Institute’s data portal. We randomly split the whole-slide images (WSIs) into 839 training slides and 210 testing slides, excluding 5 slides due to poor quality or damage. In the preprocessing phase, the WSIs were segmented into 256×256 pixel patches at two different magnifications, 20× and 10×, ensuring that each patch harbors sufficient detail for subsequent feature extraction and classification. To refine the data input and mitigate the impact of background noise, we filtered out patches containing less than 35% tissue area. Aligning with the methodologies employed in prior studies M and N, we adopted a pre-trained ResNet18 neural network as the feature extraction mechanism, converting each patch into a 512-dimensional feature vector. The choice of ResNet18 is justified by its balanced depth and proficiency in capturing intricate features, which facilitates the derivation of a rich set of feature vectors from the raw patches. This procedure provides a robust basis for the learning phase of our model.

### 4.2. Implementation Details and Evaluation Metrics

In the model training phase, the Adam optimizer was utilized for the adjustment of network weights, with an initial learning rate set at 0.0001 and a weight decay rate of $1\times 10^{-5}$ to combat overfitting. Furthermore, a cosine annealing strategy was employed to modulate the learning rate progressively throughout the training epochs. An early stopping mechanism was integrated into the training regimen to avert futile expenditures of time on suboptimal training iterations and to facilitate the rapid convergence to peak performance. For the multiple instance learning (MIL) model, each batch was composed of a single “bag”, with the training epochs totaling 50. All experimental procedures were executed on a high-capacity computing infrastructure, augmented with a cutting-edge RTX 4090 GPU.

Due to the class imbalance present in the datasets under investigation, the area under the curve (AUC) was selected as a pivotal performance metric, complementing accuracy (ACC) and the F1 score across all experimental trials. AUC is less prone to the effects of class imbalance, thereby offering a more holistic assessment of the model’s classification prowess. ACC was computed with a decision threshold of 0.5. To ascertain the consistency and dependability of the evaluation outcomes, a 10-fold cross-validation scheme was adopted, ensuring that each sample was afforded the opportunity to serve as part of the validation set. This approach permitted an equitable assessment of the model’s overall performance. Finally, the average performance metrics derived from the 10 validation subsets were reported, providing a comprehensive evaluation of the model’s overall efficacy.

We conducted a comparative analysis of the performance of our CAMIL approach on the Camelyon16 and the TCGA Lung Cancer dataset against several prominent multiple instance learning (MIL) methodologies. In pursuit of a thorough evaluation, we chose a suite of emblematic MIL techniques for benchmarking: (1) mean-pooling and max-pooling, instance-level aggregation methods that directly consolidate instance-level features to derive bag-level representations; (2) ABMIL [18], a quintessential attention-based MIL framework that assigns differential weights to instances via an attention mechanism; (3) DSMIL [20], utilizing non-local attention pooling to refine the detection of salient instances; (4) TransMIL [33], a transformer-based architecture that harnesses the transformer’s inherent strength in modeling global dependencies; and (5) CLAM, which integrates a clustering constraint mechanism into the multiple instance learning process to enhance instance feature selectivity and diversity, thereby boosting the model’s discriminative capability. The performance metrics for all comparative models were obtained using their official implementations, with hyperparameter configurations matching those of our CAMIL model. This methodological consistency ensures the evaluation’s precision and equity, enabling us to accurately gauge the performance enhancements offered by CAMIL in relation to current state-of-the-art MIL techniques.

### 4.3. Comparison and Analysis of Experimental Results

Table 1 and Table 2 delineate the experimental outcomes, revealing that under varying magnification levels, MIL methods employing max-pooling and mean-pooling techniques generally achieve an accuracy (ACC) and area under the curve (AUC) of no more than 65%. This observation further underscores the superiority of embedding-based strategies over their instance-based counterparts. A critical insight derived from the analysis of the Camelyon16 dataset indicates that in the majority of positive whole-slide images (WSIs), the regions harboring tumor cells are typically minute, accounting for less than 10% of the image area. This characteristic poses a significant challenge for most MIL frameworks, as they struggle to learn discriminative bag-level features for precise classification. Consequently, the performance of models on this dataset constitutes a stringent assessment of their capability to address such complexities.

In this challenging context, our CAMIL model demonstrates exceptional flexibility and classification precision. Notably, at a 20× magnification, CAMIL substantially outperforms other state-of-the-art models in terms of ACC, AUC, recall, and F1 score, with respective enhancements of 1.51%, 1.13%, 3.75%, and 2.71%. Furthermore, CAMIL exhibits the lowest standard deviation in AUC, recall, and F1 score, affirming its superior performance not only in terms of accuracy but also in terms of reliability and practicality for real-world applications. The experimental results at a 10× magnification similarly validate the excellence of CAMIL, with its ACC, AUC, and F1 score achieving the highest values, increasing by 0.75%, 2.84%, 1.19%, and 0.59%, respectively, compared to other top-performing models.

Additionally, we conducted supplementary experiments on the TCGA Lung Cancer dataset, as presented in Table 3. This dataset is characterized by its high heterogeneity and diverse sample sources, with significant genetic and epigenetic differences observed across subtypes such as lung adenocarcinoma and lung squamous cell carcinoma. Moreover, issues like sample imbalance and potential technical noise further complicate its clinical application. In this challenging context, the DAMIL model demonstrated remarkable competitiveness and adaptability. Consistent with its performance on the Camelyon16 dataset, DAMIL also excelled in this experiment, particularly in terms of stability, achieving leading results in accuracy (ACC), area under the curve (AUC), specificity, and recall, which underscores its robustness.

In this new experimental setting, DAMIL not only maintained outstanding performance but also exhibited exceptional adaptability to diverse pathological image data. These findings further validate our conclusion that DAMIL is a powerful tool for handling imbalanced and complex pathological image datasets. The results highlight not only DAMIL’s efficiency when utilizing slide-level labels but also its broad potential for applications in the field of pathological image analysis.

The comparative performance across different magnification levels and datasets illustrates the robustness of CAMIL in handling highly imbalanced datasets. These results not only demonstrate CAMIL’s effectiveness in addressing individual lesion labels but also indicate its potential for broader applications in pathological image analysis.

To further validate the practicality of the CAMIL method in real-world applications, we conducted an experimental comparison of its computational complexity and parameter count, with the results presented in Table 1. Although CAMIL demonstrates relatively higher computational complexity and parameter count compared to other state-of-the-art models, this cost is justified by its significant performance improvements. The advantages observed in classification accuracy (ACC), area under the curve (AUC), and other key metrics indicate that while this design introduces a certain level of computational overhead, the resulting performance gains are sufficient to offset the resource demands. Therefore, we believe that CAMIL strikes an effective balance between precision and practicality, making it a promising solution worthy of broader adoption.

The strength of the CAMIL framework lies in its highly optimized processing pipeline and modular design. Initially, the framework performs an equal-dimensional encoding of the input feature vector matrix, ensuring the efficient retention of feature information. Subsequently, it refines the matrix through the synergistic interaction of spatial attention and channel attention modules. The spatial attention module effectively identifies and enhances the spatial distribution of key instances, while the channel attention module focuses on extracting salient information from feature channels, thereby accurately modeling the intricate relationships between deep features and instances. This dual attention mechanism not only enhances the robustness of feature representation but also significantly improves the model’s ability to capture critical information. Finally, a decoder reconstructs the encoded features to produce highly accurate predictive classification results. This pipeline design not only enhances classification performance but also supports the model’s interpretability, enabling it to excel in complex tasks such as medical image analysis. Furthermore, the modular structure of the CAMIL framework offers excellent scalability, allowing it to flexibly adapt to diverse task requirements and laying a solid foundation for subsequent research.

**Figure 3:**
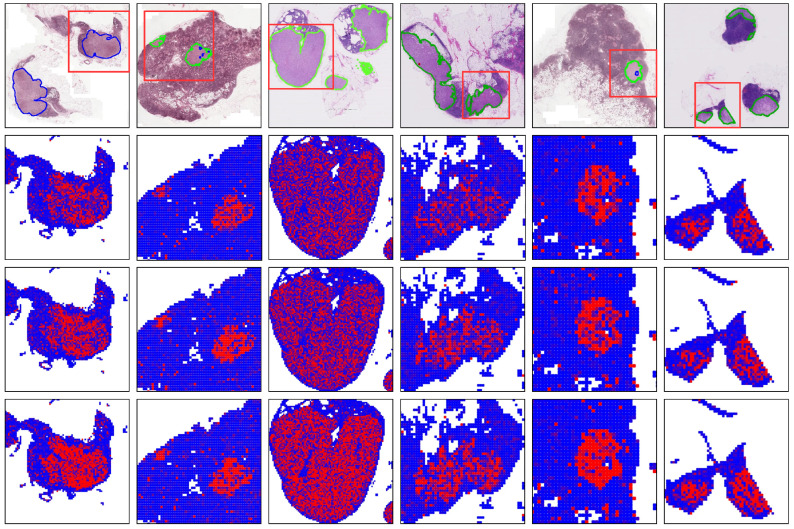
The interpretable heatmaps of the whole-slide images (WSIs) are presented. The first row illustrates the pixel-level annotations for lymph node metastasis within the WSIs, while the subsequent rows depict the corresponding interpretable heatmaps for the red-boxed regions in the WSIs, as obtained through ABMIL [18], DSMIL [20], and CAMIL, respectively. The color intensity in the heatmaps represents the confidence level of the model, with warmer colors (e.g., red) indicating a higher probability that the region is classified as a positive area, while cooler colors (e.g., blue) indicate a lower probability of being classified as a positive area.

To augment the comprehensibility of the outcomes, we integrate the grad-CAM technique into our analysis. This method elucidates the role of each instance in the network’s predictive process by assigning activation values to them. After normalizing these values, we map them accurately back to their respective spatial positions within the original whole-slide image (WSI), resulting in coherent visual representations. Figure 3 illustrates the outcomes of a comparative analysis, where the top row exhibits the pixel-level annotations of the WSI and the following three rows depict the interpretable heatmaps produced by the ABMIL, DSMIL, and CAMIL models at corresponding WSI locations.

A discerning review of these visualizations reveals that the CAMIL model not only identifies a higher number of positive instances but also demonstrates enhanced accuracy in demarcating the boundaries between tumor and normal tissue. The striking alignment between the heatmaps generated by CAMIL and the pixel-level annotations is particularly impressive. This congruence not only underscores CAMIL’s superior capability in capturing nuanced details but also highlights its potential utility in medical image processing, particularly in the precise delineation of tumor margins.

Furthermore, the Grad-CAM technique holds significant practical value in clinical applications. First, it can serve as an auxiliary diagnostic tool, providing intuitive heatmaps to help clinicians quickly locate lesion areas, thereby improving both diagnostic efficiency and accuracy. Second, Grad-CAM enhances the interpretability of AI-driven decisions by allowing healthcare professionals to verify whether the model focuses on anatomically or pathologically relevant regions, thus increasing trust in the model’s predictions. Additionally, this technique has educational potential, offering clear visual references for trainees to better understand disease characteristics and diagnostic criteria. Finally, in multidisciplinary collaborations, the visual information provided by Grad-CAM facilitates communication among experts from different fields, such as oncologists and surgeons, enabling them to jointly develop more precise treatment plans based on the highlighted regions. Therefore, this detailed visual analysis not only affirms the efficacy of the CAMIL model but also equips the medical community with a powerful tool to enhance diagnostic precision and interpret model-driven decisions.

### 4.4. Ablation Study

In this segment, we present an exhaustive narrative of a range of ablation studies performed on the Camelyon16 dataset. These investigations involve the isolated deployment of two distinct attention modules, an examination of their combined usage, as well as an evaluation of the contributions of other modules integral to the framework. Subsequent subsections will delve into the intricacies of these experimental procedures and their findings.

To validate the efficacy of the cascaded attention fusion module, we meticulously crafted and conducted a series of stringent experiments. These tests were not only focused on evaluating the module’s standalone performance but also on elucidating its synergistic benefits when integrated with other modules. Moreover, a comprehensive examination was undertaken to investigate the performance discrepancies stemming from various module combination approaches. Empirical evidence indicates that the incorporation of both channel and spatial attention modules, in contrast to the use of a single module, leads to a marked enhancement in performance, as illustrated in Table 4.

Regarding the impact of different attention fusion strategies on performance, our study reveals that prioritizing the spatial attention module followed by the channel attention module yields superior results, particularly in terms of AUC and recall metrics. This performance improvement can be attributed to the spatial attention module’s ability to first focus on critical regions within an image, thereby facilitating the precise identification of subtle or anomalous features—a capability that is crucial for medical image analysis. This process mirrors the biological visual system’s mechanism of prioritizing regions of interest, enabling the capture of global contextual information while reducing interference from background noise. Consequently, this provides more refined inputs for the subsequent channel attention module. In deep learning models, the spatial dimension typically represents the positional relationships of features, whereas the channel dimension reflects responses to various feature types (e.g., color, texture). Therefore, this sequential approach, progressing from a macroscopic to a microscopic level of refinement, aligns well with the natural hierarchy of feature extraction. Additionally, we conducted preliminary experiments exploring multiple configurations, including processing channel information first or handling both dimensions simultaneously. The results consistently demonstrated that applying spatial attention before channel attention achieved the best performance. This may be because the spatial attention module effectively filters out redundant information, allowing the channel attention module to more efficiently identify key features while also reducing computational complexity.

In contrast, a strategy that merges attention modules in parallel exhibits improved ACC and F1-score metrics. This may be due to the parallel integration allowing for more refined feature-level adjustments, concurrently emphasizing both spatial and channel information, thus enriching the feature representation. The elevated ACC denotes an enhanced capability in differentiating between positive and negative classes, while the higher F1-score indicates a more harmonious equilibrium between false positives and false negatives, which is pivotal for classification tasks. These insights suggest that different fusion strategies alter the model’s focus and comprehension of data features, resulting in diverse performance outcomes on specific evaluation metrics.

When confronting the Camelyon16 dataset, addressing its intrinsic class imbalance is paramount. Such imbalance can introduce bias into model evaluation, disproportionately benefiting criteria that better represent the model’s performance on minority classes. In this scenario, AUC stands out as a crucial evaluation metric, as it remains unaffected by class distribution imbalances and provides a holistic assessment of the model’s classification proficiency. Consequently, we selected AUC as our primary evaluation criterion. The experimental results, which showed that the cascaded fusion strategy of prioritizing spatial attention followed by channel attention surpasses other models in AUC and recall, led us to adopt this strategy as the cornerstone of our model architecture. This approach facilitates the model’s effective capture of key spatial features and the reinforcement of channel-wise feature correlations, thereby enabling a more equitable and comprehensive evaluation on imbalanced datasets.

Our research extends beyond examining the influence of attention modules on predictive accuracy to a nuanced exploration of the encoder and decoder’s individual contributions within the overarching architecture. Table 5 illustrates that the omission of either the encoder or the decoder leads to a substantial decrement in predictive performance. Significantly, the absence of the decoder exerts a more detrimental effect, a finding corroborated by all four evaluation metrics.

The primary function of the encoder is to extract and transform critical features from the input data. These high-level features are essential for capturing complex patterns and global semantic information. In our experiments, we observed that removing the encoder caused a substantial drop in model performance, particularly in tasks requiring a deep understanding of the input data. This finding underscores the indispensable role of the encoder in ensuring that the model effectively learns and represents the core characteristics of the input data. However, if other components of the model (such as the attention mechanism and decoding unit) are well-designed, they may partially compensate for the absence of the encoder, thereby mitigating the extent of performance loss.

Meanwhile, the decoder plays a pivotal role in transforming the features processed by the attention module into final predictions. Specifically, the decoder maps internal feature representations back to an interpretable output space, such as classification labels or explainable heatmaps. Experimental results indicate that the absence of the decoder disrupts this crucial pathway, preventing the model from effectively utilizing learned features for practical prediction tasks. Consequently, compared to the removal of the encoder, the absence of the decoder has a more pronounced negative impact on model performance. This observation highlights that the decoder is not merely a simple post-processing unit but rather a vital bridge connecting the feature representations to the final predictions. Its presence is critical for achieving high-quality classification outcomes.

In summary, the encoder and decoder together form a complete end-to-end framework, where the encoder provides robust feature extraction capabilities, and the decoder further enhances the model’s expressiveness and interpretability. Their synergistic interaction enables the model to fully leverage the information contained in the input data and translate it into reliable predictions. It is precisely this close collaboration that allows the model to achieve outstanding performance in classification tasks.

## 5. Conclusions and Future Work

In this investigation, we introduce an innovative multi-instance learning (MIL) framework that incorporates a cascaded attention mechanism, which significantly boosts performance over preceding approaches on the well-established Camelyon16 dataset by enhancing the model’s representation learning abilities. The core of our methodology is the application of a cascaded attention mechanism specifically designed for MIL tasks. Our mechanism outperforms conventional single-attention models by more accurately identifying discriminative features within whole-slide images (WSIs), thus improving label prediction precision. By emphasizing the most critical instances and the most prominent features within each instance, our model not only boosts predictive performance but also enhances model interpretability.

To validate the effectiveness of our proposed framework, we carried out a series of comparative experiments against contemporary state-of-the-art methods. The empirical results resoundingly confirm the superior performance of our CAMIL model compared to other leading techniques. Furthermore, to enhance model interpretability, we employed a visualization technique to illustrate the prediction process. The visualization results suggest that our model is capable of identifying a higher number of positive instances and delineating the boundaries between tumor and normal tissues with a high degree of alignment with pixel-level annotations by pathologists. We are confident that this research represents a substantial advancement in the realm of computer-aided diagnosis technologies within computational pathology and expect that CAMIL will provide valuable insights and significant enhancements for the classification of WSI using MIL.

However, while our proposed model exhibits significant improvements in pathological image classification, it still has certain limitations. Firstly, the introduction of the cascaded attention mechanism, although enhancing the model’s ability to learn important features, also increases the model’s parameter count and computational complexity. This can lead to a significant increase in training time and may pose challenges in resource-constrained environments. Secondly, while the attention mechanism helps focus on key regions, the model may still struggle to distinguish subtle differences in highly similar pathological features, potentially affecting the precision of the classification. Additionally, the diversity and complexity of pathology images may challenge the model’s generalization capabilities, especially when encountering unseen cases or image variations. Therefore, despite the model’s strong performance on specific datasets, its stability and robustness in real-world clinical applications require further validation and refinement.

In future work, we aim to develop more efficient channel and spatial attention models, along with advanced feature aggregation techniques, to comprehensively capture both macro and micro features within WSIs. These enhancements are expected to further improve the accuracy and robustness of WSI classification predictions in subsequent studies.

## Figures and Tables

**Table 1:** The experimental results, based on the Camelyon16 dataset, demonstrate performance under 20× magnification conditions. The values following the ± symbol in the table represent the standard deviation of the experimental results. The most outstanding experimental outcomes are highlighted in bold for emphasis.

Method	Accuracy	AUC	Specificity	Recall	F1-Score	FLOPs	Params
Mean-pooling	0.6391±0.0291	0.6227±0.0559	**0.9665 ± 0.0549**	0.1500±0.1070	0.2342±0.1375	62.1 M	129.5 K
Max-pooling	0.6341±0.0334	0.5344±0.0886	0.9203±0.0913	0.2062±0.1769	0.2735±0.1965	62.1 M	129.5 K
ABMIL [18]	0.8547±0.0384	0.8438±0.0718	0.9455±0.0444	0.7188±0.1112	0.7951±0.0652	77.8 M	165.3 K
DSMIL [20]	0.8420±0.0594	0.8107±0.1146	0.9538±0.0243	0.6750±0.1553	0.7652±0.1098	116.9 M	212.7 K
CLAM [19]	0.8746±0.0521	0.9012±0.0421	0.9582±0.0393	0.7500±0.0977	0.8251±0.0644	94.4 M	189.2 K
TransMIL [33]	0.8472±0.0379	0.8642±0.0504	0.9623±0.0415	0.6750±0.0874	0.7777±0.0622	610.8 M	0.63 M
CAMIL	**0.8897 ± 0.0376**	**0.9125 ± 0.0407**	0.9583±0.0681	**0.7875 ± 0.0604**	**0.8522 ± 0.0449**	165.6 M	0.32 M

**Table 2:** The experimental results, based on the Camelyon16 dataset, demonstrate performance under 10× magnification conditions. The values following the ± symbol in the table represent the standard deviation of the experimental results. The most outstanding experimental outcomes are highlighted in bold for emphasis.

Method	Accuracy	AUC	Specificity	Recall	F1-Score
Mean-pooling	0.6367±0.0197	0.6074±0.0495	0.9915±0.0180	0.1062±0.0662	0.1833±0.1013
Max-pooling	0.6440±0.0357	0.5783±0.0836	0.8784±0.1014	0.2938±0.2021	0.3600±0.1930
ABMIL [18]	0.8346±0.0393	0.8302±0.0565	0.9498±0.0645	0.6625±0.1388	0.7561±0.0787
DSMIL [20]	0.7920±0.0842	0.7863±0.1121	0.9784±0.0217	0.5188±0.2146	0.6390±0.2177
CLAM [19]	0.8497±0.0351	0.8426±0.0646	0.9623±0.0415	0.6812±0.0622	0.7837±0.0510
TransMIL [33]	0.8019±0.0514	0.8128±0.0715	0.8739±0.0829	0.6938±0.0906	0.7372±0.0627
CAMIL	0.8572±0.0408	0.8710±0.0639	0.9706±0.0442	0.6875±0.1179	0.7896±0.0749

**Table 3:** The experimental results, based on the TCGA Lung Cancer dataset, demonstrate performance under 20× magnification conditions. The values following the ± symbol in the table represent the standard deviation of the experimental results. The most outstanding experimental outcomes are highlighted in bold for emphasis.

Method	Accuracy	AUC	Specificity	Recall	F1-Score
Mean-pooling	0.7012±0.1321	0.6531±0.0326	0.9531±0.0216	0.1736±0.1643	0.2516±0.0135
Max-pooling	0.7326±0.0253	0.6153±0.0726	0.9386±0.1563	0.2341±0.0271	0.3125±0.0716
ABMIL [18]	0.8326±0.1027	0.8512±0.0426	0.9268±0.0367	0.7325±0.2105	0.7843±0.0155
DSMIL [20]	0.8465±0.1023	0.8623±0.0582	0.9414±0.1035	0.6913±0.1862	0.7810±0.1528
CLAM [19]	0.8645±0.0736	0.8924±0.0683	0.9451±0.0474	0.7653±0.0681	**0.8362 ± 0.0578**
TransMIL [33]	0.8391±0.0634	0.8762±0.1630	0.9534±0.1046	0.6934±0.0465	0.8035±0.0726
CAMIL	**0.8835 ± 0.0293**	**0.9173 ± 0.1305**	**0.9616 ± 0.0237**	**0.7682 ± 0.1025**	0.8301±0.1623

**Table 4:** A comparative analysis of experimental outcomes from the use of individual channel attention modules and spatial attention modules, as well as their various combinations, is presented. The numerical values following the ± symbol denote the standard deviations of the experimental results, with the best-performing outcomes highlighted in bold for emphasis.

Method	Accuracy	AUC	Recall	F1-Score
Only Channel	0.8597±0.0354	0.8683±0.0458	0.6750±0.0968	0.7909±0.0614
Only Spatial	0.8597±0.0373	0.8634±0.0449	0.6812±0.0804	0.7937±0.0608
Channel + Spatial	0.8997±0.0457	0.9003±0.0497	0.7875±0.1029	0.8603±0.0699
Channel → Spatial	0.8872±0.0460	0.9028±0.0477	0.7812±0.1031	0.8447±0.0719
Spatial → Channel	0.8897±0.0376	0.9125±0.0407	0.7875±0.0604	0.8522±0.0449

**Table 5:** The comparative results of the proposed framework with and without the encoder/decoder modules are presented, with the numerical values following the ± symbol indicating the standard deviations of the experimental outcomes. The best-performing results are denoted in bold for clarity.

Method	Accuracy	AUC	Recall	F1-Score
CAMIL w/o Encoder	0.8697±0.0467	0.8743±0.0409	0.7438±0.1081	0.8180±0.0721
CAMIL w/o Decoder	0.8472±0.0414	0.8461±0.0514	0.6438±0.1104	0.7667±0.0778
CAMIL	0.8897±0.0376	0.9125±0.0407	0.7875±0.0604	0.8522±0.0449

## Data Availability

The data used in this study are publicly available and can be accessed at https://camelyon16.grand-challenge.org for the CAMELYON16 dataset, accessed on 10 October 2023. The TCGA Lung Cancer dataset can be downloaded from https://portal.gdc.cancer.gov, accessed on 3 November 2023.
